# Neoadjuvant Chemotherapy Increases Genomic Radioresistance in Hormone Receptor–positive, Human Epidermal Growth Factor Receptor 2–Negative Breast Cancer: Implications for Radiation Sensitivity Index/Genomic-Adjusted Radiation Dose–Guided Radiation Therapy Personalization

**DOI:** 10.1016/j.adro.2026.102174

**Published:** 2026-08-19

**Authors:** Pierre Loap, Gilles Crehange, Youlia Kirova

**Affiliations:** a Department of Radiation Oncology, Institut Curie, Paris, France; b Innovative Radiotherapy and Systems Medicine Laboratory (IRIS), U1353 / UMR 9029, Institut Curie, Orsay, France; c Proton Therapy Center (CPO), Institut Curie, Orsay, France

## Abstract

**Purpose:**

Adjuvant radiation therapy (RT) improves locoregional control after breast cancer surgery, but dose prescription remains based mainly on clinicopathologic risk rather than individual tumor radiosensitivity. The radiation sensitivity index (RSI) and genomic-adjusted radiation dose (GARD) provide a framework for biologically guided radiation therapy personalization. In the neoadjuvant setting, however, radiosensitivity measured on the diagnostic biopsy may not reflect the residual disease ultimately targeted by adjuvant radiation therapy.

**Methods and Materials:**

We analyzed matched pretreatment and posttreatment RNA sequencing data from 2 public cohorts of hormone receptor–positive, human epidermal growth factor receptor 2–negative (HR+/HER2−) breast cancer treated with neoadjuvant chemotherapy. The discovery cohort included 16 matched pairs from GSE191127, and the validation cohort included 14 matched HR+/HER2− pairs from GSE309004. The RSI was computed using a 10-gene rank–based model. The ΔRSI was defined as RSI_post − RSI_pre. Immune fractions were inferred using quanTIseq, and the GARD was calculated for START B, RTOG 1005, and FAST-Forward adjuvant radiation therapy schedules.

**Results:**

In the discovery cohort, the mean RSI increased from 0.602 to 0.677 after neoadjuvant chemotherapy, with 12 of 16 patients (75%) showing a posttreatment increase. In the validation cohort, the mean RSI increased from 0.489 to 0.593 (Wilcoxon *P = .*014), with 11 of 14 patients (78.6%) showing an increase. In the pooled cohort, the mean ΔRSI was +0.089, and 23 of 30 patients (76.7%) showed an increased RSI after chemotherapy (Wilcoxon *P = .*005). Higher pretreatment M2 macrophage fraction correlated with a larger ΔRSI. The RSI changes translated into heterogeneous GARD dose modifications across fractionation schedules.

**Conclusions:**

Neoadjuvant chemotherapy is associated with a reproducible increase in RSI in HR+/HER2− breast cancer, suggesting that residual tumors may become more radioresistant at the genomic level after systemic therapy. A postneoadjuvant chemotherapy RSI warrants prospective evaluation for individualized adjuvant radiation therapy strategies.

## Introduction

Adjuvant radiation therapy improves locoregional control and cancer-specific survival after breast cancer surgery.^1^, ^2^, ^3^, ^4^ Yet, despite major advances in systemic therapy, surgery, imaging, and radiation delivery, radiation therapy dose is still largely prescribed according to clinicopathologic risk rather than individual tumor radiosensitivity. Most patients therefore receive similar dose-fractionation schedules, although the biological effect of a given physical dose may differ substantially across tumors.

This limitation has motivated the development of genomic approaches to radiation therapy personalization. The radiation sensitivity index (RSI) is a 10-gene, rank-based transcriptional signature designed to estimate tumor radiosensitivity,^5^^,^^6^ with higher RSI values indicating greater genomic radioresistance. RSI was subsequently incorporated into the genomic-adjusted radiation dose (GARD) framework, which combines tumor-specific radiosensitivity with physical dose and fractionation to estimate an individualized radiobiological effect.^7^^,^^8^ Together, the RSI and GARD represent one of the most developed frameworks for moving from uniform dose prescription toward biologically adapted radiation therapy.^9^

In hormone receptor–positive, human epidermal growth factor receptor 2–negative (HR+/HER2−) breast cancer, neoadjuvant chemotherapy is used for selected high-risk or locoregionally advanced tumors, including node-positive, high-grade, highly proliferative, or large tumors requiring downstaging for breast conservation.^10^ In these patients, adjuvant radiation therapy is delivered after chemotherapy and surgery, often in a context where residual disease remains a key determinant of locoregional risk.^11^ If RSI is to be used for radiation therapy personalization, an important question is therefore whether radiosensitivity should be assessed on the diagnostic biopsy or on the residual tumor after chemotherapy. This distinction matters because systemic therapy can reshape the tumor and its microenvironment. When a viable tumor remains in the surgical specimen, radiation therapy is directed at eradicating residual clonogenic disease that may persist despite surgery. A posttreatment RSI may therefore better estimate the radiosensitivity of residual disease than a baseline RSI measured before chemotherapy. Although longitudinal RSI changes have been described in triple-negative breast cancer,^12^ whether similar changes occur in HR+/HER2− disease remains poorly characterized.

In this study, we used matched pretreatment and posttreatment RNA sequencing data from 2 independent HR+/HER2− breast cancer cohorts to determine whether neoadjuvant chemotherapy alters genomic radiosensitivity. We further examined immune correlates associated with RSI dynamics and estimated the impact of RSI change on a patient-specific GARD across clinically relevant adjuvant breast radiation therapy schedules.

## Methods and Materials

### Study design and cohorts

We performed a retrospective transcriptomic analysis of 2 independent public data sets, including matched pretreatment and posttreatment breast tumor samples obtained before and after neoadjuvant chemotherapy.

The discovery cohort was derived from GSE191127, which includes HER2− breast cancers with paired RNA sequencing profiles, treatment annotations, and clinical outcome variables.^13^ RNA sequencing was performed on formalin-fixed paraffin-embedded tumor tissue using the Illumina HiSeq 2000 platform (Illumina, Inc., San Diego, California). The present analysis was restricted to HR+ and HER2− tumors, yielding 16 matched pairs. Pretreatment samples were diagnostic core needle biopsies obtained before systemic therapy. Posttreatment samples were surgical specimens collected at definitive surgery after completion of neoadjuvant chemotherapy. Extracted clinical variables included age, histologic grade, pathologic T stage, nodal status, chemotherapy regimen, neoadjuvant response index (NRI), and recurrence status. The NRI was analyzed as a continuous measure of pathologic response ranging from 0, indicating no response, to 1, indicating complete pathologic response.

An independent validation cohort was derived from GSE309004.^14^ This data set includes paired pretreatment and posttreatment samples from patients who did not achieve pathologic complete response after neoadjuvant chemotherapy, providing an independent cohort. RNA sequencing was performed on formalin-fixed paraffin-embedded tumor tissue using the Illumina NextSeq 500 platform. To match the biological scope of the discovery analysis, only HR+/HER2− tumors with complete paired samples were retained. HER2+, triple-negative, and unpaired cases were excluded, resulting in 14 matched HR+/HER2− breast cancer pairs. Available cohort-level clinical, pathologic, and treatment characteristics are summarized in Table E1.

### RNA sequencing data and RSI computation

For the discovery cohort, raw RNA sequencing counts were normalized as counts per million and log2 transformed. For the validation cohort, the deposited normalized expression matrix was used as provided, as the data were already transformed and the RSI algorithm is based on within-sample gene ranks^5^^,^^8^ rather than absolute expression values.

The RSI was computed independently for each pretreatment and posttreatment sample using the rank-based linear model originally described by Eschrich et al.^5^ The algorithm ranks the expression of 10 genes within each sample: AR, JUN, STAT1, PRKCA, RELA, ABL1, SUMO1, PAK2, HDAC1, and IRF1. An RSI score was then calculated as the weighted sum of these ranks using the published coefficients:

RSI = −0.009801 × rank(AR) + 0.012828 × rank(JUN) + 0.025455 × rank(STAT1) − 0.001759 × rank(PRKCA) − 0.003817 × rank(RELA) + 0.107021 × rank(ABL1) − 0.000251 × rank(SUMO1) − 0.009243 × rank(PAK2) − 0.020447 × rank(HDAC1) − 0.044168 × rank(IRF1).

For each patient, RSI_pre was defined as the RSI value computed from the pretreatment diagnostic biopsy, and RSI_post as the RSI value computed from the matched posttreatment surgical specimen. The paired change in an RSI score was calculated as ΔRSI = RSI_post − RSI_pre. Patients were classified as having increased, decreased, or stable RSI using a relative threshold of ±10% from baseline. This threshold was selected as a pragmatic descriptive boundary intended to approximate the reported technical reproducibility of RSI-based survival fraction at 2 Gy (SF2) prediction. Agreement between predicted and experimentally measured SF2 within ± 10% was achieved in only a subset of cell lines during validation of the original RSI model, whereas interlaboratory variability in clonogenic SF2 measurements has been reported to be approximately ±17%.^5^ The threshold was used to avoid overinterpreting small longitudinal fluctuations and was not intended as a clinically validated cutoff.

### RSI dynamics and validation analysis

The primary analysis evaluated the change in RSI between matched pretreatment and posttreatment samples. This analysis was first performed in the discovery cohort and then repeated in the independent validation cohort. For each cohort, we assessed the magnitude and direction of ΔRSI, the proportion of patients with increased, decreased, or stable RSI, and the relationship between RSI_pre and RSI_post to determine whether patient-level radiosensitivity ranking was preserved after chemotherapy. In the discovery cohort, additional exploratory analyses assessed the relationship between baseline RSI and pathologic response to neoadjuvant chemotherapy, as measured by the NRI, and the association between chemotherapy regimen and ΔRSI.

The discovery and validation cohorts were then combined into a pooled data set of 30 matched HR+/HER2− pairs. This pooled analysis was used to estimate the overall magnitude of chemotherapy-associated RSI change and to evaluate whether RSI dynamics were consistent across the 2 independent data sets after accounting for baseline RSI. Because regressing ΔRSI on baseline RSI can be affected by mathematical coupling and regression to the mean, given that RSI_pre is embedded in the definition of ΔRSI, a sensitivity analysis was additionally performed modeling RSI_post directly as the outcome, with RSI_pre and cohort identity as covariates.

### Tumor immune microenvironment analysis

To explore whether the baseline tumor immune contexture was associated with intrinsic radiosensitivity or chemotherapy-induced RSI score evolution, immune cell fractions were inferred from bulk RNA sequencing data using quanTIseq.^15^^,^^16^ This analysis was performed in the discovery cohort. Immune analyses were restricted to the 16 HR+/HER2− discovery patients. Pretreatment immune fractions were assessed in relation to RSI_pre and ΔRSI. Immune cell estimates included B cells, M1 macrophages, M2 macrophages, myeloid dendritic cells, neutrophils, natural killer cells, nonregulatory CD4+ T cells, CD8+ T cells, and regulatory T cells.

### Genomic-adjusted radiation dose

GARD was calculated from RSI using the linear-quadratic framework described by Scott et al.^7^^,^^8^ The patient-specific radiosensitivity parameter α was derived as

α=−ln(RSI)/2−2β,

with β fixed at 0.05 Gy⁻². GARD was then calculated as:

GARD=n×d×(α+β×d),

where n represents the number of fractions and d the dose per fraction.

For each patient, GARD_pre was calculated from RSI_pre, corresponding to the RSI score derived from the pretreatment diagnostic biopsy. GARD_post was calculated from RSI_post, corresponding to the RSI value derived from the matched posttreatment surgical specimen. ΔGARD was defined as GARD_post − GARD_pre. Thus, ΔGARD estimates the change in computed radiobiological dose associated with the chemotherapy-associated change in RSI for a fixed radiation therapy schedule. GARD_pre, GARD_post, and ΔGARD were calculated for 3 adjuvant breast radiation therapy schedules: Standardisation of Breast Radiotherapy Trial B (START-B trial; 40.05 Gy in 15 fractions of 2.67 Gy),^17^ Radiation Therapy Oncology Group Trial 1005 (RTOG 1005 trial; 48 Gy in 15 fractions of 3.20 Gy),^18^ and FAST-Forward trial (26 Gy in 5 fractions of 5.20 Gy).^19^ GARD was analyzed as a continuous derived metric. For a fixed radiation therapy schedule, GARD_pre and GARD_post doses share the same n and d, so the β-dependent term cancels exactly in their difference: $\Delta GARD\;=\;GARD\_post\;-\;GARD\_pre\;=\;-\left(n\times d/2\right)\;\times \;ln\left(RSI_{post}/RSI_{pre}\right).$ ΔGARD is thus proportional to $ln(RSI_{post}/RSI_{pre}),\;$with total physical dose (n × d) as the sole schedule-dependent scaling factor. Consequently, rank-based and paired statistics applied to ΔGARD are mathematically equivalent across schedules differing only in total dose.

### Statistical analysis and reproducibility

Continuous variables were summarized using means and standard deviations, or medians and ranges when appropriate. Paired pretreatment and posttreatment comparisons were performed using Wilcoxon signed-rank tests. Correlations were assessed using Spearman coefficients. Intercohort heterogeneity in the pooled analysis was assessed using a linear model with ΔRSI as the dependent variable, baseline RSI, and cohort identity as covariates. Comparisons between independent groups used 2-sample tests for continuous variables and exact tests for categorical variables; chemotherapy regimen effects were explored using the Kruskal-Wallis test. Immune associations were assessed using Spearman correlation and linear regression. For GARD analyses, paired changes were evaluated separately for each radiation therapy schedule, and associations between ΔRSI and ΔGARD were assessed using Spearman correlation. All tests were 2-sided, with a significance threshold of 0.05. Analyses were performed in R version 4.5.1 (R Foundation, Vienna, Austria).

## Results

### Neoadjuvant chemotherapy is associated with increased genomic radioresistance in the discovery cohort

Across the full discovery data set of 20 matched pretreatment and posttreatment HER2− breast cancer pairs (GSE191127^13^), neoadjuvant chemotherapy was associated with an increase in RSI. The mean RSI increased from 0.573 before treatment (SD, 0.195) to 0.683 after treatment (SD, 0.199), corresponding to a mean ΔRSI of +0.110 (SD, 0.185). This change was statistically significant (Wilcoxon signed-rank testing, *P* = .026). Sixteen of 20 patients (80%) had a posttreatment RSI increase, indicating a directionally consistent shift across individual patients.

Because the full discovery cohort included biologically distinct HER2− subtypes (including 4 triple-negative breast cancers), all subsequent discovery analyses focused on the 16 patients with HR+/HER2− breast cancer. In this subgroup, pretreatment RSI values ranged from 0.110 to 0.919, with a mean of 0.602 (SD, 0.204) and a median of 0.654, indicating substantial interpatient heterogeneity in baseline genomic radiosensitivity (Fig. 1A). After neoadjuvant chemotherapy, mean RSI increased to 0.677 (SD, 0.214), corresponding to a mean ΔRSI of +0.075 (SD, 0.234). This paired change did not reach statistical significance in the HR+/HER2− discovery cohort alone (Wilcoxon *P = .*118; Fig. 1B), but the direction of effect was consistent with that observed in the full HER2− discovery cohort.

**Figure 1:**
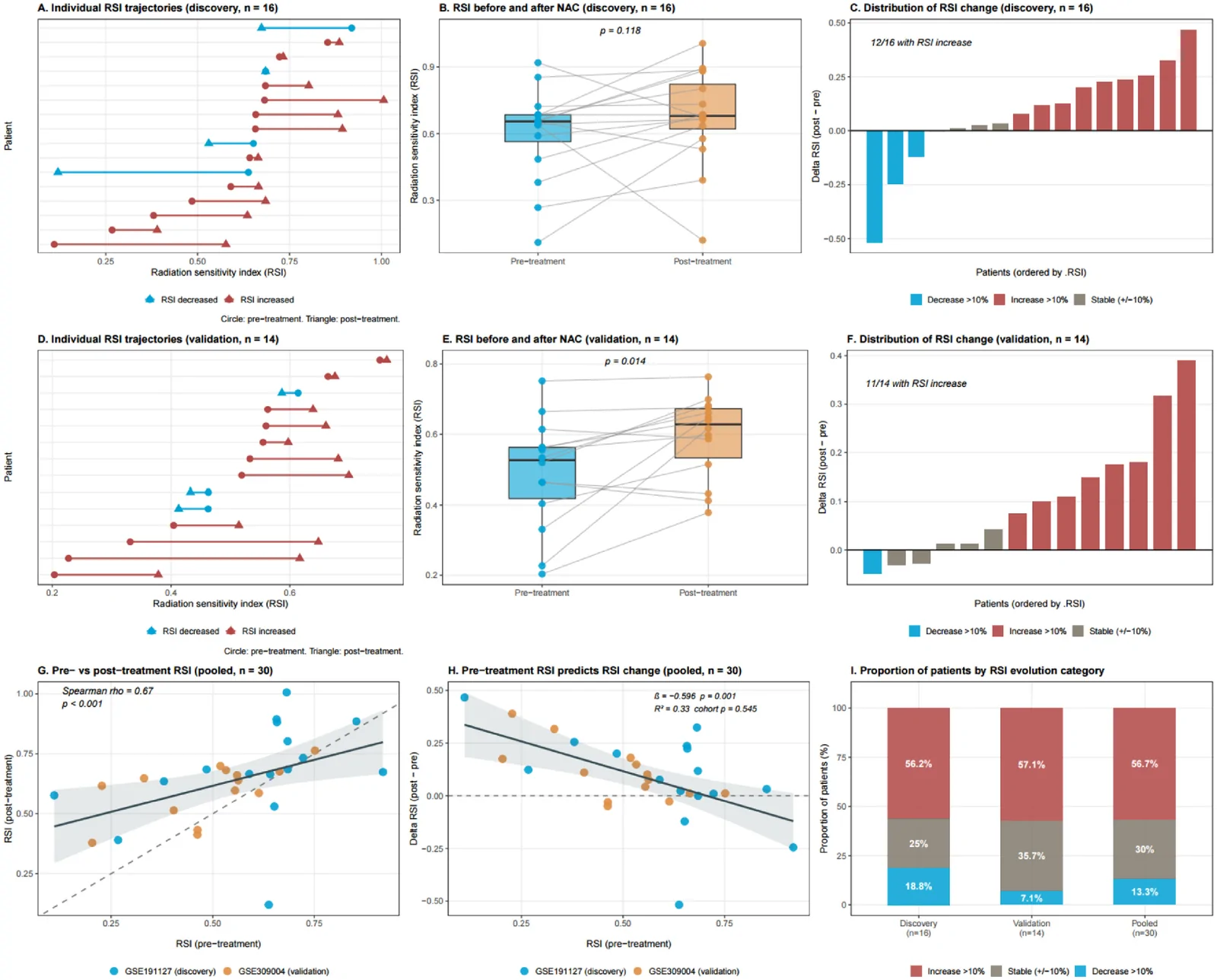
Neoadjuvant chemotherapy shifts RSI toward greater genomic radioresistance. (A) Individual pretreatment and posttreatment RSI trajectories in the discovery cohort (GSE191127, n = 16 HR+/HER2− pairs). (B) Paired RSI comparison before and after chemotherapy in the discovery cohort. (C) Waterfall plot of ΔRSI in the discovery cohort, classified using a ±10% threshold from baseline RSI. (D) Individual RSI trajectories in the validation cohort (GSE309004, n = 14 HR+/HER2− pairs). (E) Paired RSI comparison before and after chemotherapy in the validation cohort. (F) Waterfall plot of ΔRSI in the validation cohort. (G) Pretreatment versus posttreatment RSI in the pooled cohort (n = 30). A dashed line indicates no change. (H) Association between the pretreatment RSI and ΔRSI in the pooled cohort, adjusted for cohort identity. (I) Proportion of patients with increased, stable, or decreased RSI in the discovery, validation, and pooled cohorts. RSI evolution categories were defined using a ±10% relative threshold from baseline RSI. A ΔRSI was defined as RSI_post − RSI_pre. Panels B and E: paired Wilcoxon signed-rank test. Panel G: Spearman rank correlation. Panel H: linear model adjusted for cohort. *Abbreviations:* HR = hormone receptor; HER2 = human epidermal growth factor receptor 2; NAC = neoadjuvant chemotherapy; RSI = radiation sensitivity index.

At the individual level, 12 of 16 HR+/HER2− patients (75%) showed a posttreatment RSI increase, including 9 patients (56.3%) with an increase exceeding the 10% relative threshold. Four patients remained stable within ±10%, and 3 patients (18.8%) showed a decrease exceeding 10% relative to baseline (Fig. 1C). Pretreatment and posttreatment RSI values were significantly correlated (Spearman rho = 0.63, *P = .*010; Fig. E1A), indicating that the relative ordering of patients by genomic radiosensitivity was largely preserved after chemotherapy despite the overall upward shift in RSI.

Baseline RSI showed a nonsignificant inverse trend with pathologic response assessed using the NRI (Spearman rho = −0.401; *P = .*124; Fig. E1C). This result suggests a possible relationship between baseline genomic radiosensitivity and chemotherapy response, although the analysis was limited by sample size. Chemotherapy regimen did not significantly influence ΔRSI (Fig. E1B). The most frequent regimen was 6 cycles of dose-dense doxorubicin and cyclophosphamide (n = 10), with a mean ΔRSI of +0.044 (SD, 0.237).

### Independent validation supports a chemotherapy-associated RSI score increase

To validate the discovery findings, we analyzed 14 matched HR+/HER2− pairs from the independent GSE309004 cohort.^14^

Pretreatment RSI values in the validation cohort ranged from 0.204 to 0.752, with a mean of 0.489 (SD, 0.156). This baseline range was lower than that observed in the discovery HR+/HER2− cohort (mean, 0.602; SD, 0.204), consistent with differences in cohort composition and selection. After neoadjuvant chemotherapy, mean RSI increased to 0.593 (SD, 0.116), yielding a mean ΔRSI of +0.104 (SD, 0.130). This increase was statistically significant (Wilcoxon signed-rank test, *P = .*014; Fig. 1E).

Individual trajectories confirmed the directionality of the validation signal. Eleven of 14 patients (78.6%) showed a posttreatment RSI increase, including 8 patients (57.1%) exceeding the 10% relative threshold. Five patients remained stable, and only 1 patient (7.1%) had a decrease exceeding 10% relative to baseline (Fig. 1F). Individual pretreatment and posttreatment trajectories are shown in Fig. 1D. As in the discovery cohort, pretreatment and posttreatment RSI values were significantly correlated (Spearman rho = 0.56; *P = .*039; Fig. E1D), indicating preservation of patient-level radiosensitivity ranking after chemotherapy.

The validation cohort supported the discovery observation. The mean ΔRSI was numerically similar in validation and discovery cohorts (+0.104 vs +0.075), and the proportion of patients with increased RSI was nearly identical (78.6% vs 75%). The validation signal reached statistical significance despite the modest cohort size, strengthening the evidence for a chemotherapy-associated increase in RSI in HR+/HER2− breast cancer.

The discovery and validation cohorts were combined into a pooled data set of 30 matched HR+/HER2− pairs to estimate the magnitude and consistency of RSI change with greater precision. Across the pooled cohort, mean RSI increased from 0.549 before chemotherapy (SD, 0.189) to 0.638 after chemotherapy (SD, 0.177), corresponding to a mean ΔRSI of +0.089 (SD, 0.190). This increase was significant (Wilcoxon signed-rank test; *P = .*005).

At the individual level, 23 of 30 patients (76.7%) showed a posttreatment RSI increase, including 17 patients (56.7%) exceeding the 10% relative threshold. Nine patients (30.0%) remained stable, and 4 patients (13.3%) showed a decrease exceeding 10% (Fig. 1I). The distribution of individual RSI trajectories across both cohorts is shown in Fig. 1G. Pretreatment and posttreatment RSI values were strongly correlated in the pooled cohort (Spearman rho = 0.67; *P* <.001; Fig. 1G), again indicating that patient-level radiosensitivity ranking was largely maintained after chemotherapy.

To determine whether RSI dynamics differed between data sets, a linear model was fitted with ΔRSI as the dependent variable and pretreatment RSI and cohort identity as predictors (Fig. 1H). Baseline RSI was a significant negative predictor of ΔRSI (β = −0.597; SE = 0.167; *P = .*001; R² = 0.33), indicating that tumors with higher baseline RSI tended to have smaller subsequent RSI increases. In contrast, cohort identity was not associated with ΔRSI (*P = .*545). Mean ΔRSI also did not significantly differ between discovery and validation cohorts (+0.075 vs +0.104; 2-sample *t* test; *P* = .676). Because regressing ΔRSI on RSI_pre is susceptible to mathematical coupling, as RSI_pre is embedded in the outcome definition, a sensitivity analysis was performed modeling RSI_post directly as the outcome, with RSI_pre and cohort identity as covariates. RSI_pre remained a significant predictor of RSI_post (β = 0.404; *P = .*023), and the cohort effect remained nonsignificant (*P* = .545), confirming that this relationship does not reflect an artifact of mathematical coupling. Together, these findings indicate that the observed RSI increase was consistent across the 2 independent data sets after accounting for baseline radiosensitivity.

### Pretreatment M2 macrophage fraction correlates with baseline RSI and chemotherapy-associated RSI change

Immune cell deconvolution using quanTIseq^15^^,^^16^ quantified 9 immune cell populations across the 16 paired HR+/HER2− discovery samples. Pretreatment M2 macrophage fractions ranged from 0.023 to 0.129, with a mean fraction of 0.066 (SD, 0.026). After chemotherapy, the mean M2 macrophage fraction increased to 0.083 (SD, 0.032), representing a significant paired increase (t = 2.269; *P = .*038; Fig. 2A). Among the immune cell populations evaluated, the pretreatment M2 macrophage fraction showed the strongest association with baseline RSI. M2 macrophages were inversely correlated with RSI_pre, indicating that tumors with higher inferred M2 macrophage infiltration had lower baseline genomic radioresistance (β = −4.933; *P = .*009; R² = 0.398; Fig. 2B). Other immune cell populations showed weaker and nonsignificant associations with RSI_pre score (Fig. 2D).

**Figure 2:**
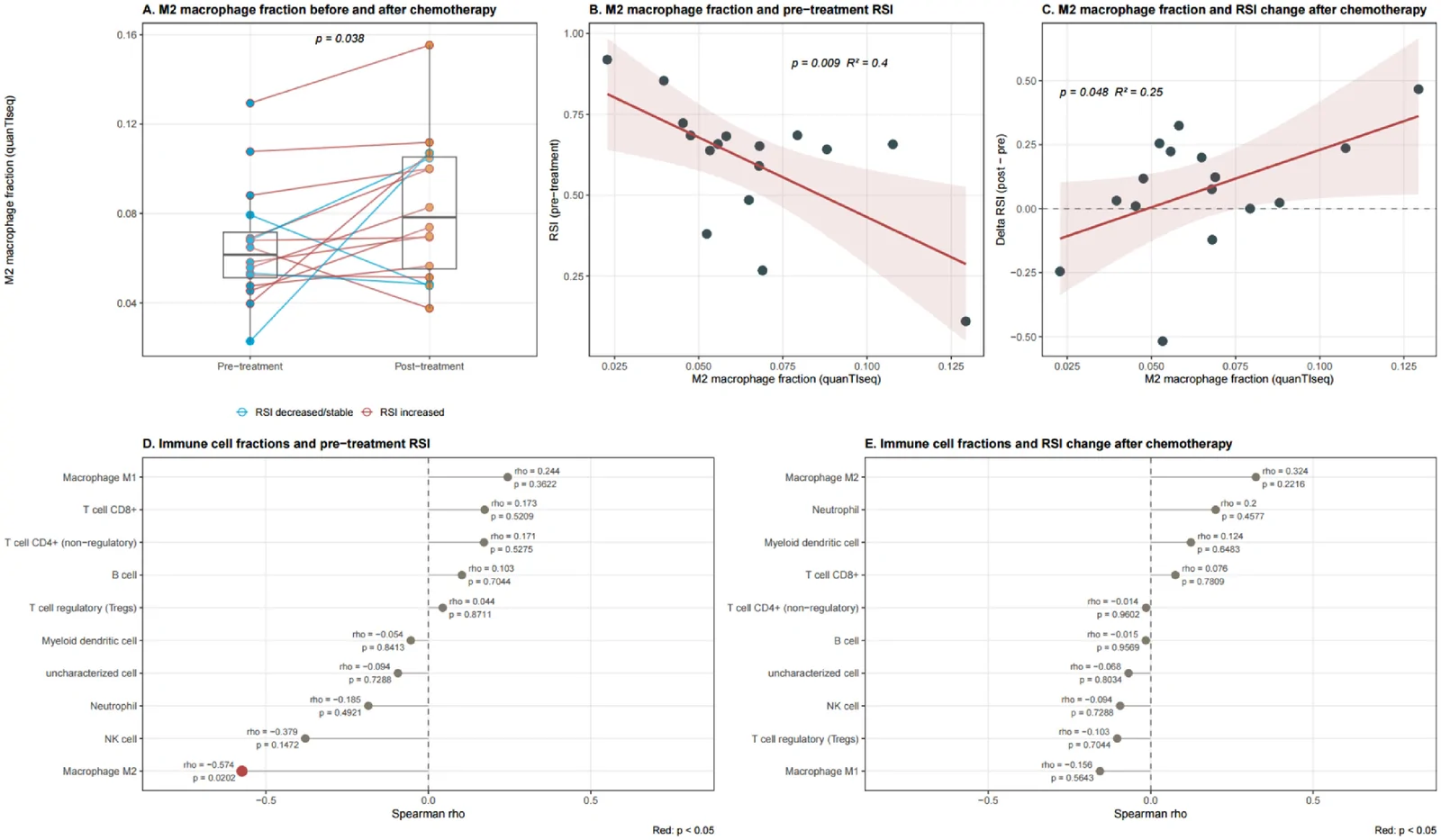
Pretreatment M2 macrophage fraction correlates with baseline RSI and chemotherapy-associated RSI change. (A) Individual M2 macrophage fraction trajectories before and after chemotherapy. (B) Association between pretreatment M2 macrophage fraction and baseline RSI. (C) Association between pretreatment M2 macrophage fraction and ΔRSI. (D) Spearman correlations between pretreatment immune cell fractions and baseline RSI. (E) Spearman correlations between pretreatment immune cell fractions and ΔRSI. Discovery cohort: GSE191127, HR+/HER2−, n = 16 matched pairs. Monocyte fraction was excluded because it was uniformly zero. Panel A: paired Student’s *t* test. Panels B and C: simple linear regression, with R² and *P* values shown in the figure. Panels D and E: Spearman rank correlation for each immune cell fraction; statistically significant associations (*P* < .05) are shown in red. *Abbreviations:* HR = hormone receptor; HER2 = human epidermal growth factor receptor 2; NK = natural killer; RSI = radiation sensitivity index.

Pretreatment M2 macrophage fraction was also associated with chemotherapy-associated RSI change using linear regression (β = +4.492; *P = .*048; R² = 0.252; Fig. 2C), although this association did not reach significance using Spearman rank correlation (rho = 0.324; *P* = *.*222), indicating limited robustness at this sample size. These results nonetheless suggest that tumors with higher inferred M2 macrophage infiltration may combine lower baseline RSI with a tendency toward larger RSI increases after chemotherapy. By contrast, aggregate macrophage estimates were not associated with either RSI_pre or ΔRSI (Spearman rho = −0.018; *P = .*952), suggesting that the observed signal was related to M2 polarization rather than total macrophage abundance. No other immune cell fraction showed a significant association with ΔRSI (Fig. 2E).

### RSI changes translate into heterogeneous GARD modifications

GARD was calculated from pretreatment and posttreatment RSI values for 3 adjuvant breast radiation therapy schedules: START-B trial (15 × 2.67 Gy), RTOG 1005 trial (15 × 3.20 Gy), and FAST-Forward trial (5 × 5.20 Gy). This analysis aimed to translate chemotherapy-associated changes in genomic radiosensitivity into patient-specific radiobiological dose estimates under clinically relevant fractionation schedules.

Pretreatment GARD varied widely across patients, reflecting baseline RSI heterogeneity (Fig. E2A). The mean pretreatment GARD was 13.37 for the START-B trial (SD, 10.44; range, 3.03-45.52), 17.30 for the RTOG 1005 trial (SD, 12.51; range, 4.91-55.83), and 11.97 for the FAST-Forward trial (SD, 6.78; range, 5.26-32.84). These wide ranges indicate that identical physical prescriptions may correspond to markedly different computed radiobiological doses across individual tumors.

Chemotherapy-associated GARD changes mirrored ΔRSI in the opposite direction, with a strong inverse correlation between ΔRSI and ΔGARD across all 3 schedules (*P* < .001; Fig. E2F). Individual GARD trajectories are shown in Fig. E2C. At the cohort level, mean ΔGARD was negative for all schedules: −2.58 for the START-B trial (SD, 12.98), −3.09 for the RTOG 1005 trial (SD, 15.56), and −1.67 for the FAST-Forward trial (SD, 8.43; Fig. E2D). None of these paired changes reached statistical significance (paired *t* test, *P = .*439 for all schedules), reflecting substantial interpatient heterogeneity in the direction and magnitude of GARD change. Because ΔGARD is mathematically equal to $ln(RSI_{post}\;score/RSI_{pre}score)$ multiplied by a schedule-specific constant proportional to total physical dose, its absolute magnitude in Gray is scaled directly with total dose across schedules (40.05, 48, and 26 Gy for START-B, RTOG 1005, and FAST-Forward trials, respectively), and the Spearman correlation between ΔRSI and ΔGARD (rho = −0.971, *P* < .001) as well as the paired *t* test *P* value (*P* = *.*439) were identical across all 3 schedules by construction, rather than reflecting independent schedule-specific findings. Pretreatment versus posttreatment GARD scatter plots (Fig. E2E) demonstrated that computed radiobiological dose decreased after chemotherapy in most patients.

These findings illustrate the rationale for integrating longitudinal genomic radiosensitivity profiling into radiation therapy personalization because the same physical dose may translate into divergent patient-specific radiobiological effects before and after neoadjuvant chemotherapy.

## Discussion

In this study, we showed that neoadjuvant chemotherapy is associated with a reproducible increase in the RSI in HR+/HER2− breast cancer. Across 2 independent matched cohorts,^13^^,^^14^ 23 of 30 patients (76.7%) showed a posttreatment RSI score increase, with a mean ΔRSI of +0.089. This increase was statistically significant in the independent validation cohort and in the pooled analysis. These findings support the central conclusion that genomic radiosensitivity, as captured by RSI, is not necessarily stable during neoadjuvant systemic therapy. Consequently, RSI measured on a diagnostic pretreatment biopsy may not fully reflect the radiobiological state of residual tumor tissue at the time adjuvant radiation therapy is delivered.

This observation is relevant because RSI is the genomic component of the GARD framework. RSI was originally developed as a rank-based systems biology model of tumor radiosensitivity^5^ and subsequently incorporated into GARD to translate genomic radiosensitivity into a radiobiological dose estimate.^7^^,^^8^ In this framework, a fixed physical dose can correspond to different biological effects depending on tumor-specific RSI. Prior clinical studies have reported associations between GARD and outcomes across tumor types, supporting its potential relevance for radiation therapy personalization, including breast cancers.^6^^,^^9^^,^^20^^,^^21^ However, most applications of RSI and GARD implicitly assume that the radiosensitivity profile measured before systemic therapy remains applicable at the time of irradiation. Our results challenge this assumption in the neoadjuvant setting by showing that RSI commonly shifts between the diagnostic biopsy and the postchemotherapy surgical specimen.

The present findings are consistent with prior work in triple-negative breast cancer showing that RSI can change during neoadjuvant therapy.^12^ Our study extends this concept to HR+/HER2− breast cancer, where adjuvant radiation therapy is frequently delivered after neoadjuvant systemic therapy and surgery for locoregionally advanced disease, yet longitudinal changes in genomic radiosensitivity remain poorly characterized. This issue is relevant in patients with incomplete pathologic response because posttreatment tumor RNA sequencing necessarily requires residual viable tumor tissue. In these patients, residual clonogenic disease may persist within or beyond the surgical bed^22^ and represents the biological target that adjuvant radiation therapy aims to sterilize. In this context, a posttreatment RSI may more closely reflect the radiobiological state of the residual disease exposed to radiation therapy than pretreatment RSI measured on the diagnostic biopsy.

A notable feature of the results is that chemotherapy shifted RSI upward while largely preserving the relative ordering of patients. In the discovery, validation, and pooled cohorts, RSI_pre and RSI_post were significantly correlated. This suggests that chemotherapy did not erase baseline interpatient differences in genomic radiosensitivity but instead induced an upward shift in many tumors while preserving much of the initial ranking. The negative association between baseline RSI and ΔRSI in the pooled model further suggests that tumors with lower baseline RSI may have greater potential for RSI increase after chemotherapy, whereas tumors with higher baseline RSI may be more stable or, in rare cases, shift toward lower RSI.

The immune analyses provide a possible biological context for these dynamics. In the discovery cohort, the pretreatment M2 macrophage fraction was inversely associated with baseline RSI, and this association was consistent across statistical approaches. A positive association between M2 fraction and ΔRSI was also observed using linear regression, suggesting that tumors with higher inferred M2 macrophage fractions may undergo larger postchemotherapy RSI score increases; however, this association was not confirmed by rank-based correlation, indicating that it is less robust and sensitive to the statistical method used. Two nonexclusive hypotheses may explain the pattern linking the M2 fraction to RSI dynamics. First, because RSI is derived from bulk RNA sequencing, M2-rich tumors may have lower baseline RSI partly through immune-derived transcriptional programs; chemotherapy-associated remodeling or depletion of this immune component could then increase RSI. Second, M2 enrichment may identify tumors in which the myeloid microenvironment is linked to greater transcriptional plasticity under chemotherapy,^23^ potentially favoring an increase in RSI in the residual tumor. Given the small sample size (n = 16), the absence of correction for multiple comparisons across the 9 immune cell fractions evaluated, and the discrepancy between statistical methods for the M2-ΔRSI score association, these findings should be viewed as hypothesis-generating rather than mechanistic proof, and the M2-ΔRSI score association in particular should be interpreted with caution pending replication in larger cohorts. Future studies using spatial or single-cell approaches will be required to determine how much of the longitudinal RSI shift reflects tumor-intrinsic radioresistance versus changes in cellular composition.

The GARD analysis illustrates the translational relevance of longitudinal RSI change. Because GARD is mathematically derived from RSI and radiation therapy fractionation,^8^ it does not provide independent biological information beyond RSI. Its value in this study is to express RSI variation in radiobiological dose terms under clinically relevant breast radiation therapy schedules. Pretreatment GARD varied widely across patients, indicating that identical physical prescriptions may correspond to markedly different estimated radiobiological effects. After chemotherapy, patients with increased RSI showed reduced GARD, whereas a smaller subgroup, particularly patients with a high baseline RSI and low baseline GARD, showed GARD gains after a decrease in RSI. The population-level RSI increase therefore conceals heterogeneous individual trajectories with opposite radiobiological implications. This supports the concept that longitudinal genomic profiling may be important if RSI or GARD is used for individualized breast radiation therapy planning.^21^

The differing absolute magnitude of ΔGARD across schedules reflects total physical dose rather than a true interaction between RSI change and dose per fraction; because β cancels in the pretreatment/posttreatment difference for any fixed schedule, ΔGARD dose is mathematically a rescaled version of the same underlying ΔRSI signal across all 3 schedules considered. An absolute GARD change was largest under the RTOG 1005 trial^18^ schedule and smaller under the FAST-Forward trial,^19^ consistent with the structure of the linear-quadratic model. This should not be interpreted as evidence that 1 fractionation schedule is clinically preferable in this data set because no radiation therapy outcome endpoint was available. Rather, it indicates that the radiobiological impact of RSI change may differ according to the physical prescription being evaluated. Importantly, a previously proposed GARD threshold of 21 was derived in triple-negative breast cancer cohorts^20^ and should not be directly applied to HR+/HER2− disease without dedicated validation.

Several limitations should be acknowledged. First, the discovery cohort was small, limiting power for secondary immune analyses. Second, both data sets were retrospective public cohorts with differences in sequencing platform and cohort composition. Third, matched pretreatment and posttreatment transcriptomic cohorts are inherently enriched for patients with residual disease because posttreatment tumor RNA sequencing requires viable tumor tissue. This feature increases the clinical relevance of the analysis for residual disease, but limits applicability to patients achieving pathologic complete response. Fourth, RSI was computed from bulk RNA sequencing without direct assessment of tumor purity or spatial cellular context.^9^ Fifth, the ±10% threshold used to define stable RSI was pragmatic and not clinically validated, particularly because the absolute magnitude of a relative change depends on baseline RSI; however, the primary analyses treated RSI as a continuous variable. Finally, no radiation therapy–specific outcome, such as local control, was available, preventing direct assessment of whether a posttreatment RSI or GARD predicts benefit from adjuvant radiation therapy.

## Conclusions

Neoadjuvant chemotherapy was associated with a reproducible increase in RSI in matched HR+/HER2− breast cancer samples, suggesting that residual tumors may become more genomically radioresistant after systemic therapy. This challenges the assumption that an RSI measured on the diagnostic biopsy reflects the radiosensitivity of the disease ultimately targeted by adjuvant radiation therapy. Prospective studies should determine whether a posttreatment RSI can better identify patients with residual radioresistant disease who may require individualized radiation therapy strategies after neoadjuvant chemotherapy.

## Declaration of AI and AI-Assisted Technologies in the Writing Process

During the preparation of this manuscript, the authors used Claude (Anthropic) to assist with language editing and refinement of the text. The authors reviewed and edited all AI-assisted content and take full responsibility for the content of the publication.

## Disclosures

The authors declare that they have no known competing financial interests or personal relationships that could have appeared to influence the work reported in this paper.

## Appendix Supplementary materials

5 - Supplementary Figure 1 Supplementary Figure S1. Additional RSI analyses. (A) Pre-treatment versus post-treatment RSI in the discovery cohort. (B) ΔRSI by chemotherapy regimen in the discovery cohort. (C) Association between baseline RSI and neoadjuvant response index. (D) Pre-treatment versus post-treatment RSI in the validation cohort, colored by luminal subtype. (E) Pre-treatment versus post-treatment RSI in the pooled cohort, colored by cohort. (F) Association between ΔRSI and neoadjuvant response index in the discovery cohort. Dashed lines in scatter plots indicate no change where applicable. Correlations were assessed using Spearman coefficients. Panels A, D, and E: Spearman rank correlation between pre-treatment and post-treatment RSI. Panels C and F: Spearman rank correlation with the neoadjuvant response index. Image, application 1

6 - Supplementary Figure 2 Supplementary Figure S2. RSI changes translate into heterogeneous GARD modifications across radiotherapy schedules. (A) Pre-treatment GARD distribution by radiotherapy schedule. (B) Post-treatment GARD distribution by schedule. (C) Individual GARD trajectories before and after chemotherapy. (D) ΔGARD by schedule. (E) Pre-treatment versus post-treatment GARD by schedule. Dashed line indicates no change. (F) Association between ΔRSI and ΔGARD by schedule. GARD was calculated using the linear-quadratic model with β = 0.05 Gy⁻² for START-B, RTOG 1005, and FAST-Forward schedules. ΔGARD was defined as GARD_post minus GARD_pre. GARD was analyzed as a continuous metric. Discovery cohort: GSE191127, HR+/HER2−, n = 16 matched pairs. Panel D: paired Student’s t-test (p = 0.439 for all three fractionation schedules, reflecting the mathematical dependence of ΔGARD on ΔRSI when fractionation is fixed). Panel F: Spearman rank correlation between ΔRSI and ΔGARD. Because ΔGARD is a linear rescaling of ΔRSI by a schedule-specific constant proportional to total physical dose (see Methods), the statistical relationship between ΔRSI and ΔGARD (Panel F) and the significance of paired changes in GARD (Panel D) are mathematically identical across schedules; only the absolute scale (Gy) differs, in proportion to total dose. Image, application 2

4 - Table 1 Image, application 3

## Footnotes

Supplementary material associated with this article can be found in the online version at doi:10.1016/j.adro.2026.102174.
