# Supplementary material for: Neoadjuvant Chemotherapy Increases Genomic Radioresistance in Hormone Receptor–positive, Human Epidermal Growth Factor Receptor 2–Negative Breast Cancer: Implications for Radiation Sensitivity Index/Genomic-Adjusted Radiation Dose–Guided Radiation Therapy Personalization
Source: Adv Radiat Oncol. 2026 Aug 19;11(12):102174. doi: 10.1016/j.adro.2026.102174 (PMC13698056; doi:10.1016/j.adro.2026.102174)
Supplement: Supplementary file 3 [file mmc3.docx]

**Supplementary Table 1. Clinical characteristics of the discovery and validation cohorts**

| **Characteristic** | **Discovery cohort, GSE191127 n = 16** | **Validation cohort, GSE309004 n = 14** |
| --- | --- | --- |
| **Dataset and sequencing** |  |  |
| Dataset | GSE191127, Hoogstrat et al. | GSE309004, Guevara-Nieto et al. |
| Sequencing platform | RNA sequencing, Illumina HiSeq 2000 | RNA sequencing, Illumina NextSeq 500 |
| Tissue type | FFPE tumor tissue | FFPE tumor tissue |
| Post-treatment sample context | Surgical specimen after neoadjuvant chemotherapy, non-pCR cases | Surgical specimen after neoadjuvant chemotherapy, non-pCR cases |
| **Pathological features** |  |  |
| Histological grade 1 | 2 (12.5%) | Not available |
| Histological grade 2 | 13 (81.3%) | Not available |
| Histological grade unknown | 1 (6.3%) | Not available |
| Pathological T1 | 1 (6.3%) | Not available |
| Pathological T2 | 13 (81.3%) | Not available |
| Pathological T3 | 1 (6.3%) | Not available |
| Pathological T4 | 1 (6.3%) | Not available |
| Node-negative disease | 6 (37.5%) | Not available |
| Node-positive disease | 10 (62.5%) | Not available |
| Disease recurrence | 2 (12.5%) | Not available |
| **Neoadjuvant chemotherapy regimen** |  |  |
| 6 cycles dose-dense AC | 10 (62.5%) | Not available |
| 3 cycles AC + 3 cycles CD | 2 (12.5%) | Not available |
| 3 cycles dose-dense AC + 2 cycles CD | 1 (6.3%) | Not available |
| 3 cycles dose-dense AC | 1 (6.3%) | Not available |
| 3 cycles dose-dense AC + 4 cycles paclitaxel | 1 (6.3%) | Not available |
| 3 cycles dose-dense AC + 9 cycles paclitaxel | 1 (6.3%) | Not available |

**Abbreviations:** HR+/HER2−, hormone receptor-positive, HER2-negative; pCR, pathological complete response; AC, doxorubicin and cyclophosphamide; ddAC, dose-dense doxorubicin and cyclophosphamide; CD, cyclophosphamide and docetaxel; FFPE, formalin-fixed paraffin-embedded.
