# Supplementary material for: Neoadjuvant Chemotherapy Increases Genomic Radioresistance in Hormone Receptor–positive, Human Epidermal Growth Factor Receptor 2–Negative Breast Cancer: Implications for Radiation Sensitivity Index/Genomic-Adjusted Radiation Dose–Guided Radiation Therapy Personalization
Source: Adv Radiat Oncol. 2026 Aug 19;11(12):102174. doi: 10.1016/j.adro.2026.102174 (PMC13698056; doi:10.1016/j.adro.2026.102174)
Supplement: Supplementary file 5 [file mmc2.docx]

# Supplementary Figure 2
